# Ethical and scientific issues of gene-edited twin by clustered regularly interspaced short palindromic repeats Cas9 technology

**DOI:** 10.4274/jtgga.galenos.2019.2019.0153

**Published:** 2020-06-08

**Authors:** Esra Bilir, Emine Elif Vatanoğlu Lutz, Mustafa Levent Özgönül

**Affiliations:** 1 Koç University Faculty of Medicine, İstanbul, Turkey; 2 Department of Medical History and Ethics, Koç University Faculty of Medicine, İstanbul, Turkey; 3 Department of Medical History and Ethics, Akdeniz University Faculty of Medicine, Antalya, Turkey

To the Editor,

In November 2018, He Jiankui, a Chinese biophysicist at Southern University of Science and Technology, announced through an online video that the first gene-edited twin girls were born without chemokine receptor type 5 (CCR5). This had been achieved by using clustered regularly interspaced short palindromic repeat (CRISPR)-Cas9 technique and was undertaken because the father was infected with human immunodeficiency virus (HIV). The aim was to create HIV resistant offspring. Although the goal of creating humans with HIV resistant genes seems very reasonable and needs to be appreciated, he did not receive any plaudits but the contrary. This was because he had acted against a number of the fundamental principles of medical practice including “First do no harm”, the Nuremberg Code which states that one must not conduct research on humans without animal experiment, and the Helsinki Declaration (HD) concerning human enhancement and intervention in the human embryo (1). The HD is the main ethical document of the World Medical Association, which regulates the ethical norms of all clinical research in all fields globally (1).

This endeavour was criticized by the authorities around the World because gene-edited human embryos using CRISPR-Cas9 has never been approved to reach birth because of the unpredictable side effects, and also because the technique is still imperfect (2). The first question that should be answered by he is how he managed to start and complete this “experiment” on human embryos without any form of ethical committee approval. The weakness of institutional board control and national legal regulations regarding the use of gene-editing technologies in humans might have enabled him to perform this “experiment”. However, he acted against basic international rules of scientific research, especially those involving humans. Although he is not a medical doctor, the team must have included a gynecologist to perform oocyte pick-up and this doctor should have informed the authorities and also prevented he from contacting the couple claiming to be the parents of the twins. It remains unclear whether the team obtained informed consent by fully explaining the consequences and possible unpredictable side effects of using CRISPR-Cas9.

There are also major scientific limitations to CRISPR-Cas9 technology. It is not 100% effective for inserting new gene(s) or deleting gene(s) and also might lead to unwanted mutations that are potentially harmful to humans (3). More specifically some scientists have reported concerns about neurological deficits after knockdown of CCR5 by CRISPR-Cas9 (4). The twin girls were claimed to be free of the CCR5 gene; however, it is known that CCR5 is not the only route that HIV enters cells. Since HIV is not only physically and psychologically debilitating, but culturally and socially devastating too, this case can be considered as a promising option for people infected with HIV (5). They have the right to reproduce and have healthy offspring. Nevertheless, this should be achieved by well-established methods, not by experimental and possibly harmful technologies.

There is an urgent need for legal regulations to control the usage of CRISPR-Cas9 in humans, that both international and national authorities should prepare and adhere to. In Turkey all regulations are based on the HD. We believe that it is very important that all innovative clinical research fields should first be harmonized according to the ethical standards of the HD.
